# Inability to rescue stalled ribosomes results in overactivation of the integrated stress response

**DOI:** 10.1016/j.jbc.2024.107290

**Published:** 2024-04-16

**Authors:** Ankanahalli N. Nanjaraj Urs, Victor Lasehinde, Lucas Kim, Elesa McDonald, Liewei L. Yan, Hani S. Zaher

**Affiliations:** Department of Biology, Washington University in St Louis, St Louis, Missouri, USA

**Keywords:** ribosome, stalling, protein synthesis, E3 ubiquitin ligase, Hel2, mRNA decay, ribosome quality control, integrated stress response, Gcn2, Gcn4

## Abstract

Endogenous and exogenous chemical agents are known to compromise the integrity of RNA and cause ribosome stalling and collisions. Recent studies have shown that collided ribosomes serve as sensors for multiple processes, including ribosome quality control (RQC) and the integrated stress response (ISR). Since RQC and the ISR have distinct downstream consequences, it is of great importance that organisms activate the appropriate process. We previously showed that RQC is robustly activated in response to collisions and suppresses the ISR activation. However, the molecular mechanics behind this apparent competition were not immediately clear. Here we show that Hel2 does not physically compete with factors of the ISR, but instead its ribosomal-protein ubiquitination activity, and downstream resolution of collided ribosomes, is responsible for suppressing the ISR. Introducing a mutation in the RING domain of Hel2—which inhibits its ubiquitination activity and downstream RQC but imparts higher affinity of the factor for collided ribosomes—resulted in increased activation of the ISR upon MMS-induced alkylation stress. Similarly, mutating Hel2’s lysine targets in uS10, which is responsible for RQC activation, resulted in increased Gcn4 target induction. Remarkably, the entire process of RQC appears to be limited by the action of Hel2, as the overexpression of this one factor dramatically suppressed the activation of the ISR. Collectively, our data suggest that cells evolved Hel2 to bind collided ribosomes with a relatively high affinity but kept its concentration relatively low, ensuring that it gets exhausted under stress conditions that cannot be resolved by quality control processes.

A key feature of all living organisms is their ability to respond and adapt to an ever-changing environment to ensure cellular homeostasis regardless of the conditions surrounding them. For example, they are adept at responding to nutrient availability, limiting or increasing their proliferation in response to nutrient-poor or -rich conditions, respectively (1). In response to chemical or physical stress, organisms activate a variety of processes, including quality control mechanisms that preserve the integrity of biological molecules as well as stress response pathways enabling them to mitigate the effect of damage. Shared among all organisms is also their ability to quickly reprogram gene expression to adapt and/or to survive changing environmental conditions (2, 3, 4, 5, 6, 7, 8, 9).

Notably, in response to most environmental insults, organisms may activate multiple pathways that have distinct consequences downstream. This can be partly explained by the presence of multiple sensors that can recognize the very same signal. Therefore, the activation of the appropriate pathway critically depends on the correct sensor recognizing the signal. Often when the same primary sensor is activated, the intensity and/or the duration of the signal can lead to different outcomes (4, 6, 10). Illustrating this point is how cells respond to damaging agents that compromise the integrity of their biological molecules: on one hand, they promptly activate repair and/or quality control pathways, on the other hand, if the damage persists, they arrest the cell cycle and/or induce apoptosis (11, 12, 13). How and when cells switch between these responses is highly regulated, involving many sensor and effector molecules.

Since the process of translation is a highly energetic one, the ribosome has long been appreciated to be a target for many signaling processes (7, 14). For example, in response to nutrient availability, the TOR (target of rapamycin) pathway phosphorylates several translation factors and ribosomal proteins, which in turn promote protein synthesis (1). Conversely, in response to stress, the integrated stress response (ISR) is activated leading to inhibition of protein synthesis (5). Remarkably, in addition to being a target of these pathways, the ribosome has recently emerged as a central sensor for them. In particular, it seems that organisms have taken advantage of the fact that the elongation phase is exquisitely sensitive to environmental conditions, and evolved factors to recognize stalled ribosomes to activate a number of pathways (4, 7, 15, 16, 17, 18, 19, 20).

Ribosome stalling can be induced in response to various stress conditions, including amino-acid depletion and chemical insults that damage RNA (6, 21, 22, 23). Interestingly, ribosome stalling often leads to collisions, and it is the collided ribosomes that appear to signal for downstream quality-control and stress-response pathways (7, 24). During ribosome quality control (RQC), collided ribosomes are recognized by the E3 ubiquitin ligase Hel2 (ZNF598), which ubiquitinates ribosomal proteins uS3, uS10 and eS7 (16, 17, 25, 26, 27). uS10 ubiquitination signals for RQC to initiate, ribosome disassembly by the RQT (RQC-trigger) complex, and degradation of the nascent peptide (16). Both uS10 and eS7 ubiquitination signal for degradation of the defective RNA through no-go decay (NGD) (25). It is worth noting that the initiation of RQC and NGD is completely dependent on the ability of Hel2 to recognize and ubiquitinate ribosomes (8, 25, 28). In addition to RQC, recent evidence suggests that the ISR is also activated by collided ribosomes (4, 6). Briefly, the ISR is a conserved eukaryotic pathway activated in response to biotic and abiotic stresses. The process is characterized by phosphorylation of the translation initiation factor eIF2α, which leads to global inhibition of translation. Notably, phosphorylated eIF2α leads to selective translation of pro-survival genes that include *GCN4* in yeast (*ATF4* in humans). Gcn4 and ATF4, are transcription factors and the key effectors of the ISR; they induce the transcription of hundreds of genes, including those involved in amino-acid synthesis (29, 30, 31). In mammals, four eIF2α kinases exist, GCN2, PERK, PKR, and HRI, responding to amino-acid deprivation, ER stress, dsRNA viral infection, and cytoplasmic protein misfolding, respectively (7, 31). GCN2 appears to be the ancestral kinase for all four, as it is found in all eukaryotes and the only one eIF2α kinase found in yeast (32). Initial studies on yeast Gcn2 revealed that the factor is activated by deacylated tRNAs, which accumulate when amino acids become limiting, using its His-tRNA synthetase (HisRS) like domain (21, 22, 33, 34). More recent work from several groups, including ours, has shown the factor to be more broadly activated under different stress conditions by recognizing stalled ribosomes (4, 6). Indeed, a cryo-EM structure showed that its coactivator Gcn1 binds collided ribosomes (10). Therefore, it appears that both Hel2 and Gcn2’s coactivator can recognize collided ribosomes under the same conditions that cause translational stalls.

Importantly, the choice of which of these factors is recruited to the ribosomes has important consequences downstream for which biological pathway is activated. Emerging from multiple studies is that the initiation of these pathways is intricately coordinated to ensure the appropriate one is activated (7). For instance, we recently showed that the initiation of RQC on collided ribosomes seems to be more robust than that of the ISR, and its activation suppresses Gcn2-mediated eIF2α phosphorylation (6). Notably, the molecular mechanics underlying this apparent competition are not fully understood. In this study, we used genetic manipulations of yeast to address this question. Generation of a catalytically dead Hel2 variant results in overactivation of the ISR under collision-inducing conditions, even with observed increased binding by the mutant factors to collided ribosomes. Similarly, inhibition of RQC independently of Hel2 by introducing mutations in uS10 that stop its ubiquitination increases the accumulation of Gcn4 and transcription of its targets. Our data reveal that RQC and the ISR factors do not appear to directly compete with each other for binding on colliding ribosomes. Instead, inhibition of the ISR activation by Hel2 is a consequence of downstream RQC activity of ribosome disassembly. Finally, overexpression of Hel2 was found to significantly repress Gcn2 activation, suggesting that RQC activity is severely limited by the availability of the factor. Our findings imply that yeast may have fine-tuned Hel2 levels to ensure that the ISR is activated when the damage exceeds a critical threshold.

## Results

### Mutations in the RING domain of Hel2 inhibit NGD on stalling reporters irrespective of the nature of the stall

**Figure 1:**
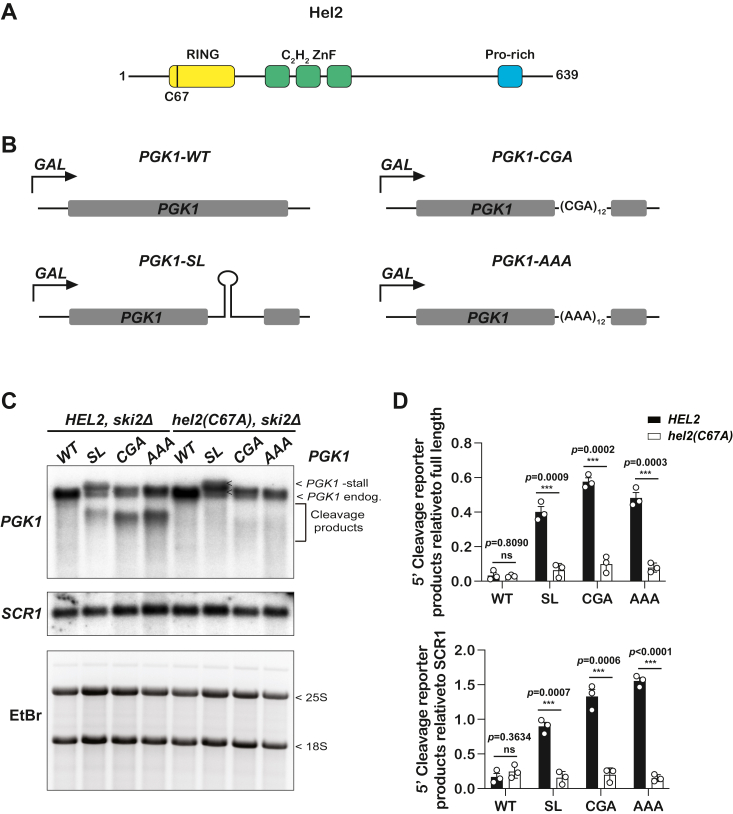
**C67A mutation in the RING domain of Hel2 impairs no go decay on stalling reporters.***A*, schematic showing the domain architecture of Hel2 protein, including its N-terminal RING domain, middle 3 × C_2_H_2_ Znf domains, and the C-terminal proline-rich domain. The location of the C67A in the RING domain is shown. *B*, schematics of the control and three stalling *PGK1* reporters used to follow NGD. *C*, phosphor images of northern analyses of 5′ cleavage fragments in the indicated cells harboring the depicted *PGK1* reporter. Shown are representatives of three replicates. *D*, bar graphs are used to display the quantification of northern blots like those shown in (C). Plotted are the relative intensities of the 5′ fragments to those of the full-length product (*top*) or to the intensities of *SCR1* (*bottom*) in the indicated cells having the shown reporter. In all cases, plotted are the means from three independent biological replicates, with the error bars representing the standard deviation around them. Significance calculated with a two-tailed, unpaired *t* test. ∗∗∗- *p* < 0.001; ∗∗- *p* < 0.01; ∗- *p* < 0.05; ns, not statistically significant.

Hel2, a key sensor of collided ribosomes, contains an N-terminal RING domain followed by three C_2_H_2_-type zinc finger (ZnF) domains and a C-terminal proline-rich domain (Fig. 1*A*) (25). Previous data have shown that the RING domain is essential for NGD and RQC to be initiated on stalling reporters (25, 35). Before assessing how mutations in this domain impact induction of the ISR, we sought to establish that in our yeast background, mutation of the conserved cysteine residues of the ZnF within the RING domain inhibits NGD. We substituted cysteine 67 with an alanine (C67A) and introduced a C-terminal FLAG tag to the factor. For our control strain, we only introduced the tag, which allowed us to detect the wild type and mutant factor. We used an integrating plasmid containing the tagged *HEL2* variants along with its native regulatory regions; promoter, 5′-UTR, and 3′-UTR (655 bp upstream and 225 bp downstream). The two plasmids were introduced to the *ADE2* locus in *hel2Δ* cells. To assess the effect of the RING-domain mutation on NGD, we used a collection of *PGK1* reporters based on one originally described by Parker and colleagues (36). These reporters, in which *PGK1* is under a *GAL* promoter, harbor structural or sequence elements that stall elongation by the translating ribosome (Fig. 1*B*) (17). These include a stable stem-loop (SL) (5′ -GAT ATC CCG TGG AGG GGC GCG TGG TGG CGG CTG CAG CCG CCA CCA CGC GCC CCT CCA CGG GAT ATC-3′), a stretch of the inhibitory poly-Arg codons (PGK1-(CGA)_12_), and a poly A tract encoding poly-Lys (PGK1-(AAA)_12_) (17, 36, 37, 38). We introduced these reporters into an exosome-deficient strain *ski2Δ*, which stabilizes the 5′-end fragments generated by NGD, allowing us to monitor the efficiency of the endonucleolytic cleavage (36). As expected, northern analysis of the expression of these reporters revealed an accumulation of shorter RNA products that were absent in the non-stalling wild-type reporter (Fig. 1*C*). More importantly, however, was the observation that the C67A mutation of Hel2 inhibited the accumulation of the truncated RNA products, consistent with earlier reports on the critical role of the RING domain during NGD initiation (25, 39). Quantification of the northern blots verified that the C67A mutation abolishes the generation of truncated RNA products from all stalling reporters, confirming the essentiality of the RING domain for NGD (Fig. 1*D*).

### Hel2 C67A mutant binds collided ribosomes with a better apparent affinity relative to the wild-type factor

**Figure 2:**
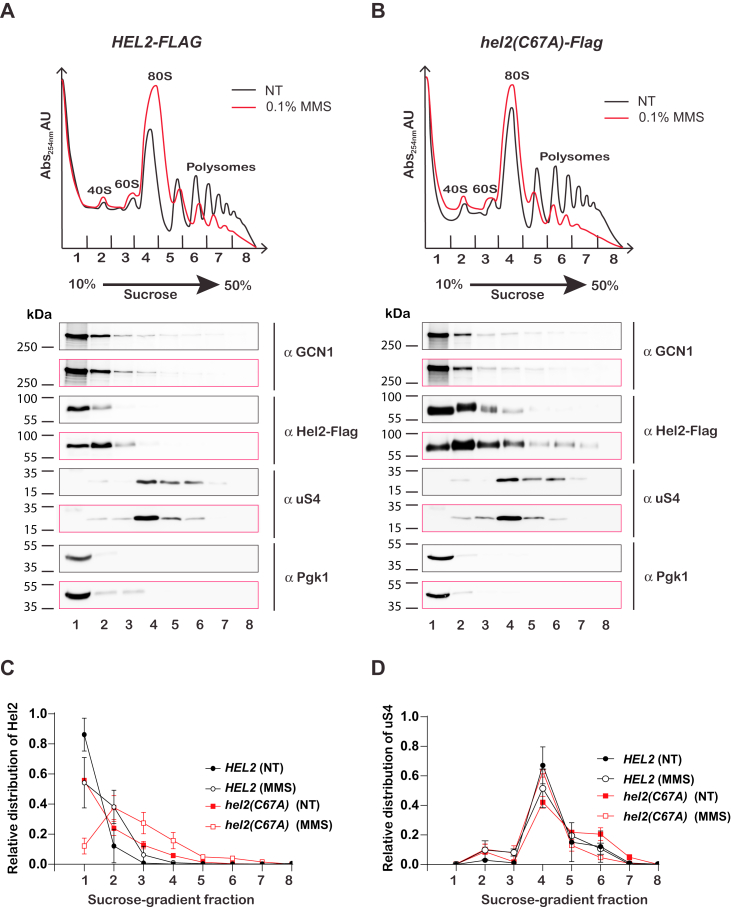
**Mutation of the RING domain results in increased binding of Hel2 to collided ribosomes.***A*, *top* shows polysome profiles of lysates isolated from cells expressing wild type Hel2 (Flag tagged) in the absence and presence of MMS. Lysates were fractionated using ultracentrifugation over 10 to 50% sucrose gradients. *Bottom* shows immunoblot analyses of fractions isolated from the same sucrose gradients with the indicated antibodies. *B*, same as in (A) but with the C67A mutant of Hel2. *C* and *D*, line graphs are used to show the quantification results of the immunoblot analyses like those in (A) and (B). The lines in (C) display the relative distribution of Hel2 in the indicated cells (wild type and C67A-mutant cells) in the absence and presence of MMS. Similarly, the lines in (D) display that of ribosomal protein uS4 in the same cells and under the same conditions. In all cases, the average values from three independent experiments are plotted, and the error bars represent the standard deviations.

**Figure 3:**
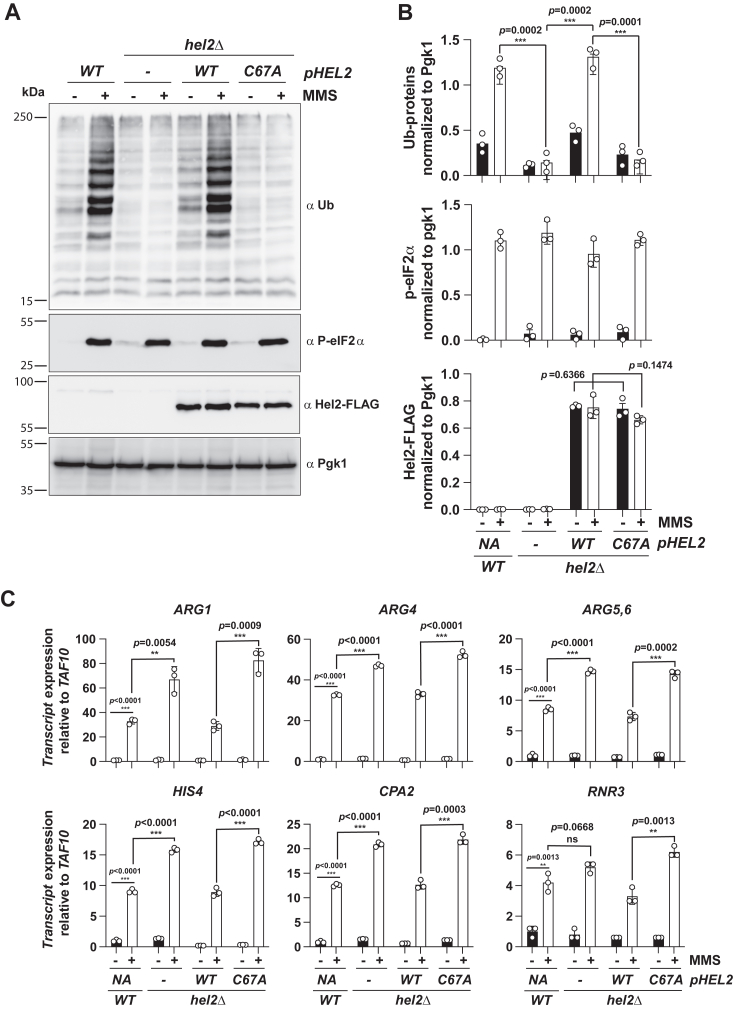
**The C67A mutation of Hel2 leads to overactivation of the ISR in the presence of MMS.***A*, representative immunoblot analyses of lysates isolated from the indicated cells that were cultured in the absence and presence of MMS. Membranes were probed with the indicated antibodies. *B*, quantification of immunoblots like those shown in A. Bar graphs are used to show the relative intensity of ubiquitinated proteins (*top*), phosphorylated eIF2α (*middle*) and Hel2 (*bottom*). In all sets of graphs, the intensities were normalized to those of Pgk1. *C*, bar graphs used to summarize the results of qRT-PCR analysis of the indicated transcripts relative to *TAF10* in the shown cells that were grown in the absence and presence of MMS. For all graphs, plotted are the means quantified from three biological replicates, with the error bars describing the standard deviations around them. Significance calculated with a two-tailed, unpaired *t* test. ∗∗∗- *p* < 0.001, ∗∗- *p* < 0.01, ∗- *p* < 0.05, ns-not statistically significant.

The observation that the C67A mutation in Hel2 inhibits NGD can in principle be rationalized by multiple experimental complications, including decreased stability of the protein and/or its inability to bind ribosomes. The first of these appears unlikely based on earlier reports (16, 25) and data in this study (Figs. 2 and 3). To address the second scenario, we conducted sucrose-gradient ultracentrifugation to fractionate lysates isolated from wild-type and C67A mutant cells grown in the absence and presence of 0.1% MMS for 30 min. The addition of MMS damages RNA and promotes widespread ribosome stalling and collision (6, 40, 41). We used immunoblot analysis to assess the association of Hel2 with ribosomes and polysomes (Fig. 2). As expected, wild-type Hel2 migrated with the light fractions of the gradient and slightly migrated down the gradient when cells were grown in the presence of MMS (Fig. 2, *A* and *C*). Interestingly, the C67A mutant migrated with the 40S, 60S, and 80S subunits even when cells were untreated. For samples treated with MMS, the mutant migrated deep into the gradient, where polysomes are found (Fig. 2, *B* and *C*). Immunoblotting for the ribosomal protein uS4 and Pgk1 was used to confirm that the fractionation worked as intended (Fig. 2, *A* and *B*). These observations reveal that the RING-domain mutant of Hel2 associates better with collided ribosomes relative to the wild-type factor and suggest that ubiquitination of ribosomal protein decreases the affinity of the factor for ribosomes.

### The RING-domain mutant of Hel2 results in overactivation of the ISR

Having established that the C67A mutation in Hel2 inhibits NGD without impacting its ability to bind ribosomes, we next asked whether it alters the induction of the ISR. To monitor the process, we used MMS to trigger alkylation stress, ribosome stalling, and activation of RQC and Gcn2. Indeed, the addition of MMS resulted in increased ribosomal-protein ubiquitination and eIF2α phosphorylation (Fig. 3, *A* and *B*). As expected, the deletion of *HEL2* abolished the accumulation of the ubiquitinated polypeptides upon the addition of MMS (Fig. 3, *A* and *B*). Complementing the *hel2Δ* cells with a wild-type copy of the factor restored the ubiquitination signature seen in the *HEL2* cells. By contrast, the reintroduction of the C67A mutant did not, phenocopying the null mutant (Fig. 3, *A* and *B*). Immunoblotting for Hel2 confirmed that the mutation has no effect on the stability of the protein. These observations suggest that the inability of Hel2 to ubiquitinate stalled ribosomes sensitizes cells to stress, over-inducing Gcn2.

To quantify the effect of the RING-domain mutation on induction of the ISR, we conducted quantitative reverse transcription PCR (qRT-PCR) analysis of a number of Gcn4 targets. In the presence of MMS, we observed ∼30, 30, 9, 12, and nine-fold induction of *ARG1; ARG4; ARG5,6; CPA2;* and *HIS4* genes, respectively in the wild-type cells (Fig. 3*C*). Deletion of *HEL2* resulted in a further ∼twofold increase in their levels. Complementation with the native gene restored the induction of all these genes to wild-type levels (Fig. 3*C*). This was in direct contrast to what we measured when the *hel2Δ* cells were complemented with the C67A mutant, which exhibited increased induction levels for all the Gcn4 targets, similar to those observed in the non-complemented cells (Fig. 3*C*). In summary, our qRT-PCR analyses revealed that inhibition of the ubiquitination activity of Hel2 results in the overactivation of the ISR.

### The inability to induce RQC through uS10 ubiquitination is responsible for the hyperactivation of the ISR

**Figure 4:**
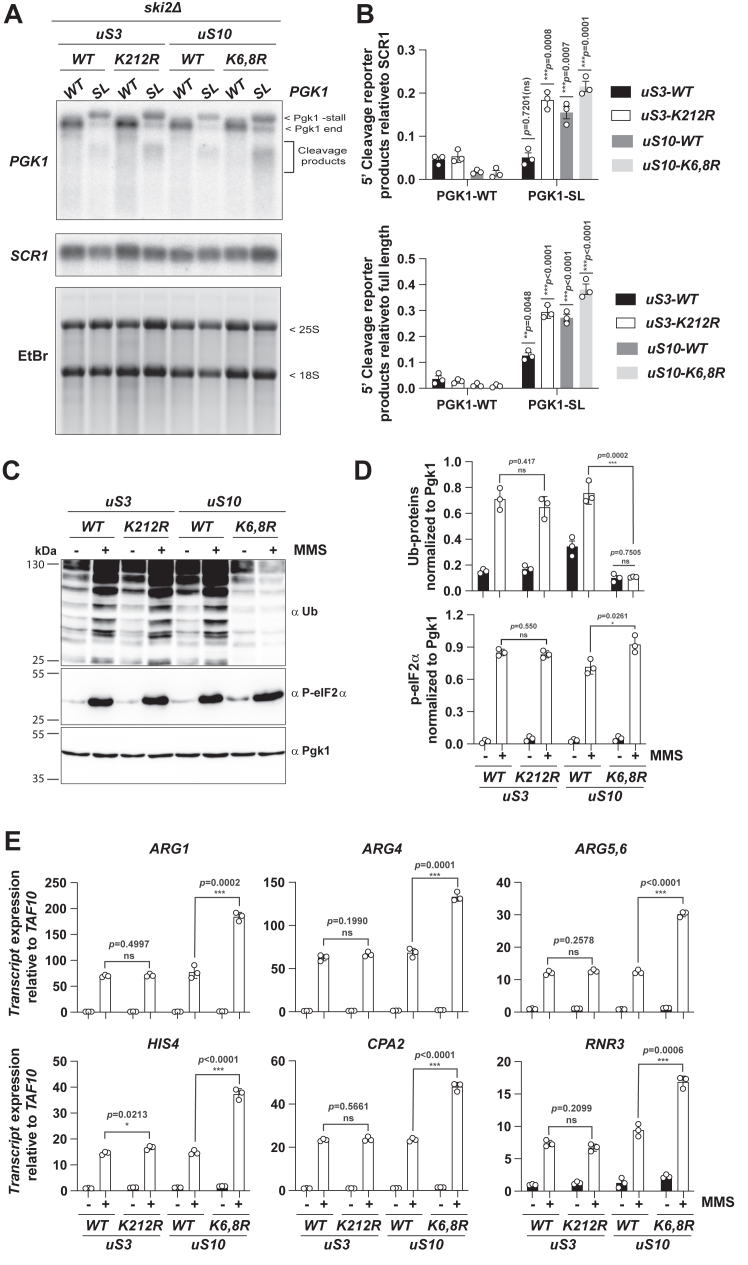
**Inhibition of ubiquitination of uS10and not that of uS3 causes overactivation of the ISR.***A*, Northern-blot analyses of the indicated cells transformed with WT or SL *PGK1* reporters. Membranes were probed with *PGK1-* and *SCR1*-specific probes. The bottom is an image of the gel stained with EtBr prior to transfer. Shown are representative images of three biological replicates. *B*, quantifications of the relative intensity of *PGK1*’s 5′ fragments in the indicated cells harboring the shown reporter. The top shows a bar graph, for which the intensities were normalized to *SCR1’s* intensities; for the bottom one, the intensities were normalized to the full-length *PGK1* product. *C*, immunoblot analyses of lysates collected from the indicated strains in the absence and presence of MMS. *D*, bar graphs describing the quantification results of the relative levels of ubiquitinated proteins (*top*) and phosphorylated eIF2α (*bottom*) from immunoblot analyses like those shown in (*C*). In both cases, the signals were normalized to those of Pgk1. *E*, bar graphs are used to show the qRT-PCR analyses of the relative abundance of the indicated transcripts to *TAF10* in the indicated cells in the absence and presence of MMS. In all cases, plotted are the means from three independent biological replicates, with the error bars representing the standard deviation around them. Significance calculated with a two-tailed, unpaired *t* test. ∗∗∗- *p* < 0.001, ∗∗- *p* < 0.01, ∗- *p* < 0.05, ns, not statistically significant.

On the ribosome, Hel2 has multiple ribosomal-protein substrates, with each serving a distinct signaling purpose (7, 16, 25). In particular, the protein is known to add ubiquitin chains to at least three ribosomal proteins: uS3, eS7, and uS10. Ubiquitination of uS3 has a role during nonfunctional ribosome decay (NRD) (42, 43), that of eS7 has a role in CCR4-Not mediated co-translational mRNA decay, and that of uS10 is critical for the initiation of RQC (8, 16, 44, 45). As a result, we next addressed which of these ubiquitination events are important for the induction of the ISR. We and others have mapped the lysine residues on each of these proteins that are ubiquitinated in response to stress (16, 25, 27, 40, 43). In particular, Hel2 adds ubiquitin chains on K212 of uS3, K63 of eS7, and K6 and K8 of uS10. Notably, eS7 is encoded by two paralogues, and deletion of either paralogue before our attempt to introduce mutations on the other paralogue resulted in decreased polysome levels and compromised induction of the ISR. As a result, we focused our mutational analysis on uS3 and uS10. More specifically, we mutated K212 of the native uS3 protein to an Arginine (K212R). On the native uS10, we introduced K6R and K8R mutations. Both uS3 and uS10 mutants were introduced into *ski2Δ* cells to monitor NGD on our stalling reporter. We note that Ikeuchi *et al*. previously reported that NGD can proceed through an alternative pathway when uS10 ubiquitination is inhibited. This alternative NGD^RQC-^ pathway is dependent on eS7 ubiquitination and leads to multiple cleavage reactions further upstream of the stalling sequence, resulting in the production of a heterogenous population of cleavage intermediates (25). In complete agreement with these earlier reports, K212R mutation in uS3 had no noticeable impact on the accumulation of the 5′-cleavage fragments in the presence of the PGK1-SL reporter as assessed by northern analysis of its transcript (Fig. 4, *A* and *B*). By contrast, northern analysis of the same reporter in the presence of the uS10 K6,8R mutant revealed the production of heterogenous 5′-cleavage intermediates seen as a smear on the blot (Fig. 4, *A* and *B*).

We next assessed the effect of these mutations on the activation of Gcn2. Similar to Hel2 mutants, we carried out immunoblot analysis to evaluate MMS-mediated ribosomal-protein ubiquitination and eIF2α phosphorylation on uS3 and uS10 mutants. Consistent with the idea that uS10 ubiquitination is critical for RQC, mutation of its lysine substrates resulted in a complete loss of MMS-induced ubiquitination signal on our immunoblots (Fig. 4, *C* and *D*). In contrast, mutation of the lysine substrates in uS3 appeared to have no effect on the same ubiquitin signal (Fig. 4, *C* and *D*). To add further support for these observations that Gcn2 is hyperactivated when uS10 ubiquitination is inhibited, we performed qRT-PCR analysis of Gcn4 target induction in the presence of MMS. Similar to what we observed for the Hel2 RING-domain mutant, MMS-mediated induction of Gcn4 targets was more than twofold higher in the presence of uS10 ubiquitination-dead mutant (Fig. 4*E*). Conversely, no changes to the levels of the same transcripts were observed in the uS3 mutant (Fig. 4*E*). Altogether, our data suggest that the inability of cells to ubiquitinate uS10 during ribosome stalling and initiate downstream RQC hyperactivates Gcn2 and subsequent induction of the ISR.

### Overexpression of *HEL2* results in suppression of Gcn2 activation

**Figure 5:**
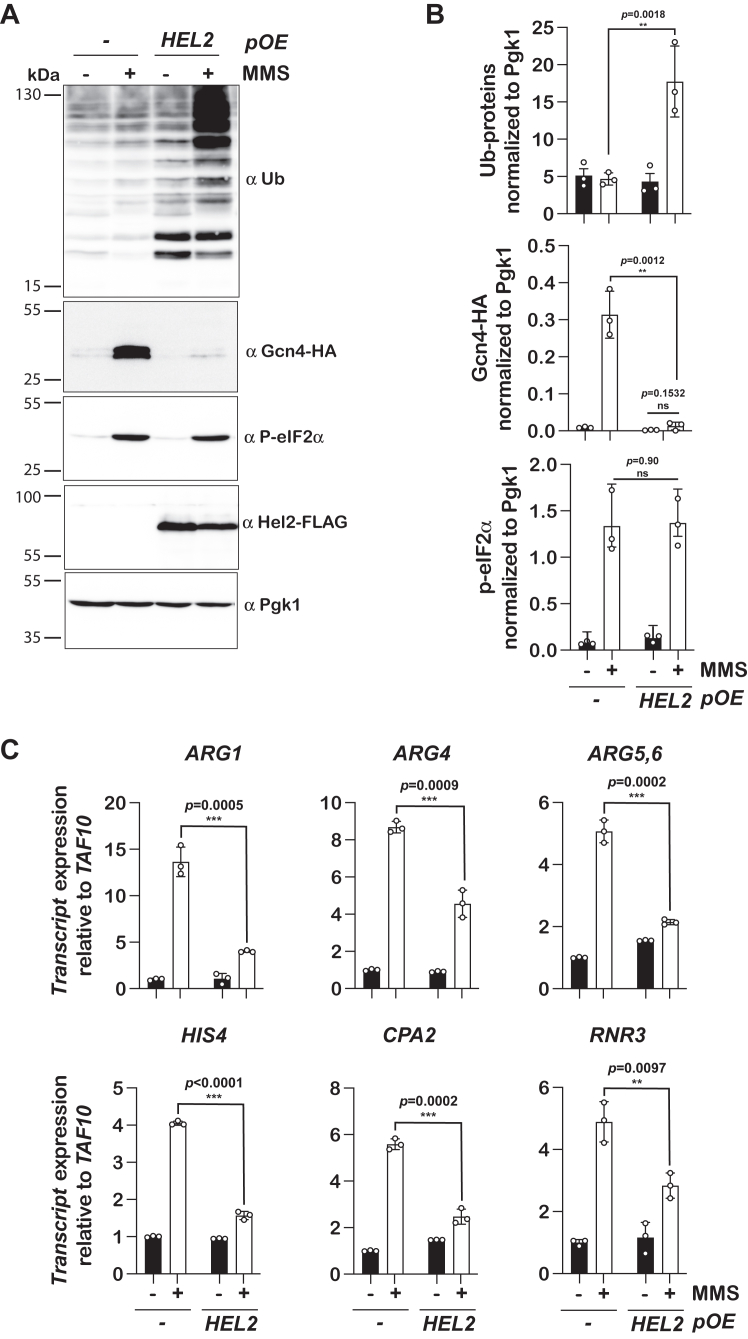
**Overexpression of Hel2 suppresses the activation of the ISR.***A*, representative immunoblot analyses of lysates collected from cells expressing an empty vector or Hel2 cultured in the absence and presence of MMS. *B*, bar graphs describing the quantification results of immunoblots like those shown in (*A*). The *top*, *middle* and *bottom* plots show the results for the relative levels of ubiquitinated proteins, Gcn4, and phosphorylated eIF2α, respectively. In all cases, the intensities of the particular protein products were normalized to those of Pgk1. *C*, bar graphs used to summarize the qRT-PCR analyses of the relative levels of the indicated transcripts to *TAF10* in cells transformed with the indicated plasmids in the absence and presence of MMS. For all bar graphs, plotted are the means of three biological replicates, and the error bars represent the standard deviations around them. Significance calculated with a two-tailed, unpaired *t* test. ∗∗∗- *p* < 0.001, ∗∗- *p* < 0.01, ∗- *p* < 0.05, ns, not statistically significant.

RQC is a multi-step process, initiating with Hel2-mediated ribosomal ubiquitination, followed by mRNA decay and dissociation of the two subunits before the peptidyl-tRNA-bound large subunit is resolved (46). Our data so far suggest that inhibition of the entire process results in overactivation of Gcn2, but they do not provide details about which of the process’s above steps might be restrictive of the induction of the ISR. Given its low cellular concentration estimated to be ∼2000, *i.e.*, about 1% of the number of ribosomes (47, 48), we suspected that ubiquitination of ribosomal protein by Hel2 is limiting for the process. To address this possibility, we overexpressed the factor using a high-copy plasmid and assessed its effect on Gcn2 activation. Immunoblot analysis of cells overexpressing Hel2 revealed a significantly decreased accumulation of Gcn4 in the presence of MMS relative to cells harboring an empty vector control (Fig. 5, *A* and *B*). In agreement with these observations, qRT-PCR analysis showed an approximately threefold decrease in the induction of the Gcn4 targets when *HEL2* was overexpressed (Fig. 5*C*). Our analyses, therefore, greatly suggest that Hel2-mediated ubiquitination during ribosome stalling serves as a bottleneck for the RQC process.

## Discussion

Our data presented here provide some important mechanistic insights into the interplay between the activation of RQC and the ISR in response to ribosome stalling. We have previously shown that these pathways are in apparent competition with each other (6). These initial analyses hinted that factors in the two pathways do not physically compete for the same site on ribosomes, but provided no data that addressed these proposals directly. In this study, we tackled these unsettled ideas and presented compelling evidence suggesting that the RQC collision sensor Hel2 does not physically compete with the ISR factors on the ribosome. Instead, the ability of the factor to ubiquitinate uS10, and hence initiate RQC, appears to be critical for its role in antagonizing the activation of the ISR. In particular, by generating a RING-domain mutant of the factor, which binds ribosomes with better affinity (Fig. 2), we showed that mere binding of Hel2 to collided ribosomes is insufficient to suppress the activation of Gcn2 (Fig. 3). Indeed, mutation of the lysine substrates in uS10, whose ubiquitination is critical for downstream events of RQC, resulted in overactivation of Gcn2 (Fig. 4) even though Hel2 binding is not affected. These observations are in agreement with a model that Hel2-mediated ribosome rescue decreases the concentrations of collided ribosomes, which are required for induction of the ISR. In other words, by clearing collided ribosomes, Hel2 prevents Gcn2 and its coactivators from ever interacting with them in the first place. We expanded on these ideas by presenting data that suggest Hel2 is the limiting factor for RQC, and in turn suppression of premature activation of Gcn2. In particular, we show that overexpression of Hel2 on its own is sufficient to suppress the activation of Gcn2 (Fig. 5).

Hel2 (and its human homolog Znf598) was one of the first factors to be recognized to be activated in response to ribosome collisions (16, 17, 26). Since this initial discovery, several factors have been shown to bind collided ribosomes - including Gcn1 and Gcn20, the coactivators of Gcn2; Mbf1, which prevents frameshifting during stalling; ZAKα, a MAPKKK; and cGAS, which activates the innate immune response (10, 18, 20, 49, 50). However, even with its large area, the disome interface might get saturated with the number of factors that can bind at the same time. Since we do not have any molecular details about where Hel2 binds on this interface, it would have been reasonable to assume that it might occupy the same site as Gcn2 and/or its coactivators, preventing their activation. Our data, however, suggest that the ubiquitination of ribosomes by Hel2, and its downstream events of RQC, suppresses induction of the ISR. We provided two independent lines of evidence to support these conclusions. In the first one, mutating the RING domain of Hel2, and inhibiting its ability to ubiquitinate ribosomal proteins, increased its affinity for collided ribosomes, yet it had the same impact on the activation of Gcn2 as the null mutant. In particular, MMS-induced accumulation of Gcn4 target genes was significantly increased in the presence of the C67A mutant (Fig. 3*C*). We confirmed that this mutant completely abolished NGD and RQC, as judged by the absence of endonucleolytic cleavage products from stalling reporters and lack of MMS-induced ubiquitin signals on our immunoblots, respectively (Figs. 1*C* and 3*A*). Notably, in addition to RQC, Hel2 has been implicated in a number of processes, including NRD and mRNA decay (25, 43). Our analysis with the Hel2 mutant in principle could not distinguish between which of these processes impacts Gcn2 activation. Since the initiation of these processes is marked by a ubiquitination modification on a specific ribosomal protein, we were able to specifically inhibit each of them without having to modify the E3 ligase. In particular, mutating K6 and K8 of uS10 to arginine residues, the initial ubiquitination sites necessary for subsequent polyubiquitination that occurs on K63 of ubiquitin itself, inhibited the ubiquitination of the protein and RQC altogether, resulting in the overactivation of the ISR as evident by increased levels of Gcn4 targets, phenocopying the same effects seen in *hel2Δ* cells (Fig. 4). We note that the K6,8R mutation does not completely abolish NGD, since eS7-mediated NGD^RQC-^ can still be initiated (7, 25). In turn, these observations strongly suggest that induction of RQC and not that of NGD is important for the apparent suppression of the ISR.

**Figure 6:**
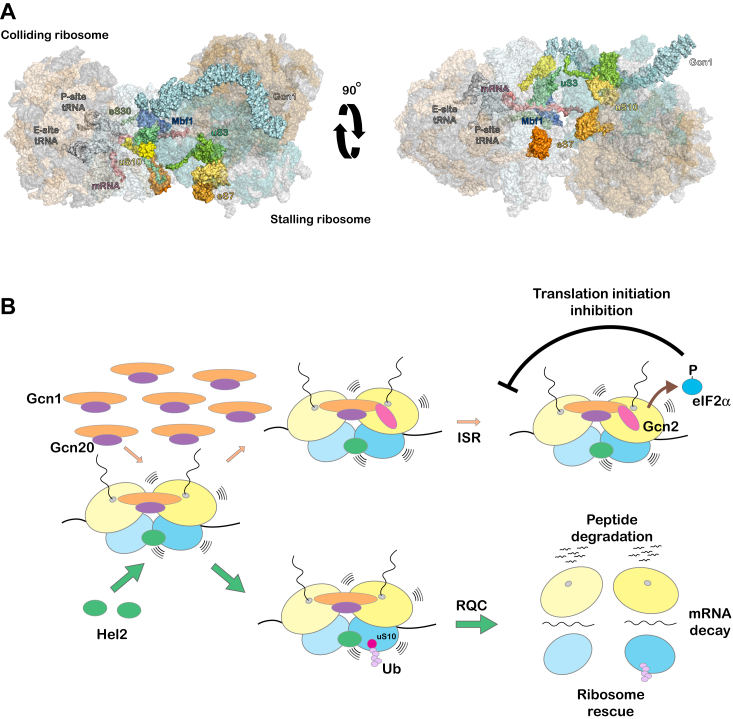
**Hel2 does not appear to compete with Gcn1 for binding on collided ribosomes.***A*, structure of Gcn1-bound collided ribosomes from (PDB 7NRC and 7NRD) (10). Highlighted are the important players, including Gcn1 and Hel2’s ribosomal protein targets. The right view shows the interface where Hel2 is likely to bind (based on the location of its substrates), which is on the opposite side of Gcn1. *B*, a model showing how the activation of RQC and the ISR might be coordinated on collided ribosomes. In this model, Hel2-mediated ubiquitination of ribosomal proteins and downstream RQC activation is more robust than Gcn1/20-mediated activation of Gcn2. The concentration of Hel2, however, is kept low, leading to its exhaustion when the damage is severe.

Except for Gcn1, and Mbf1 (and its human homologue EDF1), we have no structures of ribosomes bound to the other collision sensors (10, 18). However, our data presented here, together with what we know about its ribosomal protein substrates, may offer some clues about Hel2’s mode of binding on collided ribosomes (51). Particularly since our data revealed that RQC and the ISR do not physically compete, Hel2’s binding surface is not likely to overlap with that of Gcn1, Gcn20, and Mbf1. On collided ribosomes, Gcn1 binds in an extended conformation with its N- and C-termini interacting with the P stalks of the large subunits of the colliding and stalling ribosomes, respectively (10). Some of its middle HEAT repeats make interactions with the disome interface, making contact with eS12 and eS31 of the colliding ribosome. Remarkably, these proteins occupy an interface distinct from the one formed by Hel2’s ribosomal protein substrates of uS3, eS7, and uS10, suggesting that the binding of Hel2 is unlikely to interfere with that of Gcn1 (Fig. 6*A*). A model describing the interplay between the activity of Hel2 and Gcn1 is shown in Figure 6*B*, in which we emphasize that the downstream activity of Hel2 is responsible for RQC’s apparent suppression of the ISR. We note that in the absence of a ribosome-bound Gcn2 structure, we do not have a full understanding of how Gcn1 activates it, and especially whether Hel2’s ubiquitination activity could alter it. Clearly, more work is needed to further dissect the interplay between these two processes, and in particular, the kinetic and thermodynamic parameters that make RQC more robust at recognizing and rescuing collided ribosomes.

## Experimental procedures

### Yeast strains, plasmids, and primer

Yeast strains, plasmids, and primers are listed in supporting information Tables S1–S3, respectively.

### Plasmid construction

The plasmids encoding the PGK1 gene and PGK1-SL under the control of the GAL1 promoter were obtained from Roy Parker (36). Plasmids encoding PGK1-(CGA)_12_ and PGK1-(AAA)_12_ were made as described earlier (17). Plasmids encoding FLAG-Hel2 and FLAG-Hel2 (C67A) were generated using Gibson assembly (52) (NEB, Cat#: E2611S).

### Polysome analysis

Saturated overnight yeast cultures in YAPD media were diluted to OD_600_ 0.05 in 1 L culture. When cells reached OD_600_ 0.6 to 0.7, half of the culture was treated with 0.1% MMS (Sigma Aldrich, Cat#:129925) for 30 min to induce alkylation stress. Cells were immediately harvested by vacuum filtration and pellets were frozen in liquid nitrogen. Cell pellets were finely powdered with pestle and mortar in liquid nitrogen and resuspended in 1 ml of lysis buffer (20 mM Tris pH 7.5, 140 mM KCl, 1.5 mM MgCl_2_, 1% Triton X-100, 0.1 mg/ml cycloheximide). Supernatant from cleared lysate corresponding to 800 to 1000 μg of total RNA was layered over a 10% to 50% sucrose gradient containing 20 mM Tris pH 7.5, 150 mM KCl, 5 mM MgCl2, 500 mM DTT and centrifuged at 37,000 rpm for 160 min at 4 °C in an SW41Ti (Beckman) swinging bucket rotor. Gradients were fractionated using a Brandel tube-piercing system combined with continuous absorbance reading at A_254_ nm. Gradient fractions were TCA precipitated for protein isolation and resuspended in HU buffer (8 M Urea, 5% SDS, 200 mM Tris pH 6.8, 100 mM DTT, 1 mM ethylenediaminetetraacetic acid (EDTA), bromophenol blue) for immunoblotting.

### Western blot

For Western blot assays, mid-log-phase yeast cells were harvested and lysed in 1 ml of ice-cold lysis buffer (300 mM NaOH, 1% β-Mercaptoethanol). Proteins were then precipitated by adding TCA to 7.5% and resuspended in HU buffer (200 mM Tris pH 6.8, 8 M Urea, 1 mM EDTA, 5% SDS, 100 mM DTT, and bromophenol blue) using a volume that was normalized to the number of cells harvested. Proteins were resolved on 12% SDS-PAGE gels and transferred to PVDF membranes using a semi-dry transfer apparatus (Bio-Rad). The membranes were blocked with 5% milk in TBST for 60 min at room temperature followed by incubation with primary antibody overnight at 4 °C. After washing with TBST, the membrane was incubated with the appropriate HRP-conjugated secondary antibody for 1 h at room temperature before washing 3 to 4 × with TBST. Detection was carried out on a GE Image Quant LAS 4000 using Super Signal West Pico PLUS Chemiluminescent Substrate (Thermo Fisher Scientific, Cat#: 34580). The following antibodies were used: Mouse ANTI-FLAG HRP (Sigma Aldrich, Cat#: A8592; 1:3000 v/v dilution), mouse anti-ubiquitin HRP (Santa Cruz, Cat#: sc8017; 1:2000 v/v dilution), rabbit anti-Gcn1 (from Alan Hinnebusch lab), rabbit anti phospho-eIF2α (Ser51) (Cell Signaling Technology, Cat#: 3398S; 1:5000 v/v dilution), rabbit anti-uS4 (Rps9) (Abcam, Cat#: ab117861; 1:5000 v/v dilution), Mouse PGK1 Antibody (Invitrogen, Cat#: 459250; 1:5000 v/v dilution). Secondary antibodies of goat anti mouse IgG HRP (Thermo Scientific, Cat#: 31430) and goat anti rabbit IgG HRP (Thermo Scientific, Cat#: 31460) were used at (1:10,000 v/v dilution). Densitometry analysis was conducted using Image Quant Version 7.0 (Cytiva). Unless otherwise stated, the relative signal of the protein of interest was obtained by normalizing to Pgk1.

### Quantitative RT-PCR

Total RNA from yeast cells was isolated following the hot phenol method as described earlier (6, 53). 1 to 10 μg of total RNA that was treated with DNase I (Thermo Scientific, Cat#: EN0521) was used to generate cDNA with M-MuLV reverse transcriptase (NEB, Cat#: M0253L) using a random hexamer (Thermo Scientific, Cat#: SO142) for priming. Quantitative RT-PCR was conducted using iTaq Universal SYBR Green Supermix (Bio-Rad, Cat#: 1725121) with ∼50 ng of cDNA. The relative fold change was obtained by following the ΔΔCt method. The relative transcript abundance for each gene from three biological repeats was determined by normalizing to the expression level of the *TAF10* gene.

### Northern blotting

Yeast cells were grown overnight in a defined media (-Ura + 2% glucose). Saturated overnight cultures were washed twice with -Ura media containing 2% raffinose and 2% galactose. OD_600_ was cut down to 0.1 and allowed to grow until 0.6 to 0.7 to induce the *GAL*-driven reporters. Cells were pelleted by centrifugation, washed with AE buffer (50 mM NaOAC pH 5.2 and 10 mM EDTA) and quickly frozen on dry ice and stored in −80 °C. RNA was isolated from frozen cell pellets by hot acid phenol/chloroform extraction method as described earlier (6, 53). The samples were phenol-chloroform extracted and ethanol precipitated a second time. 5 μg of total RNA was resolved on 1.2% formaldehyde agarose gel, followed by transfer to positively charged Zeta-Probe Blotting membranes (Bio-Rad) using a vacuum blotter (Bio-Rad) following the manufacturer’s instructions for transferring RNA. Nucleic acids were UV cross-linked to the membrane (150W-2K lamp at 40 cm for 10 min) and baked at 80 °C for 15 min. After cross-linking, the membrane was placed in a glass hybridization bottle with the RNA side facing away from the glass. Approximately 13 ml of Perfect Hyb Plus Hybridization Buffer (Sigma, Cat#: H7033) was added and the bottle was placed in a hybridization oven for 60 min at 42 °C to block the non-specific probe binding sites on the membrane. Radiolabeled DNA probe, which was labeled using polynucleotide kinase (NEB, Cat#: M0201S) and [γ-^32^P] ATP (PerkinElmer, Cat#: NEG035C001MC), was added to the buffer and incubated overnight. Membranes were washed with non-stringent buffer (2 × SSC, 0.1% SDS) three times, followed by three washes in stringent buffer (0.2 × SSC, 0.1% SDS), all at hybridization temperatures for 15 min. Membranes were then exposed to a phosphor imager screen and analyzed using an Amersham Typhoon RGB imager.

### Quantification and statistical analysis

Statistical and graph analyses for Northern blots, Western blots, and RT-qPCR were processed by GraphPad Prism 8.4.3 (GraphPad Software) and expressed as mean ± SD of at least three independent experiments. The comparison between the groups was considered statistically significant only if *p* < 0.05 by Student’s *t* test (two-tailed, unpaired).

## Data availability

All data are contained within the manuscript. Additional information and requests for reagents and resources are available from the corresponding author (hzaher@wustl.edu).

## Supporting information

This article contains supporting information detailing yeast strains, plasmids (17, 36, 40, 54) and oligonucleotide primers used.

## Conflict of interest

The authors declare that they have no conflicts of interest with the contents of this article.
