# Supplementary material for: Inability to rescue stalled ribosomes results in overactivation of the integrated stress response
Source: J Biol Chem. 2024 Apr 16;300(5):107290. doi: 10.1016/j.jbc.2024.107290 (PMC11106528; doi:10.1016/j.jbc.2024.107290)
Supplement: Supporting Tables S1–S3 [file mmc1.docx]

## **Inability to rescue stalled ribosomes results in overactivation of the integrated stress response.**

Ankanahalli N Nanjaraj Urs^1^, Victor Lasehinde^1^, Lucas Kim^1^, Elesa McDonald^1^, Liewei L Yan^1^, and Hani S Zaher^1,2,*^

^1^ Department of Biology, Washington University in St. Louis, St. Louis, MO, USA 63130

^2^ Lead Contact

*Correspondence: hzaher@wustl.edu

Department of Biology

Washington University in St. Louis

Campus Box 1137, One Brookings Drive

St. Louis, MO, USA 63130

Phone: (314) 935-7662

Fax: (314) 935-4432

## **Supporting information: Tables and References**

## **Table1: Yeast Strains**

| **Yeast Strain** | **Genotype** | **Source** |
| --- | --- | --- |
| BY4741 | *MATa his3Δ1 leu2Δ0 met15Δ0 ura3Δ0* | Dharmacon lab |
| Hel2Δ | *BY4741; hel2Δ::Leu2* | This Study |
| Hel2-Flag | *BY4741; hel2Δ::Leu2, EKD-Hel2-FLAG: Met* | This Study |
| Hel2 C67A-Flag | *BY4741; hel2Δ::Leu2, EKD-Hel2 C64,67A-FLAG: Met* | This Study |
| uS3 | *BY4741; uS3-WT::His* | This Study |
| uS3-K212R | *BY4741; uS3-K212R::His* | This Study |
| uS10 | *BY4741; uS10-WT::His* | This Study |
| uS10-K6,8R | *BY4741, uS10-K6,8R::His* | This Study |
| Hel2-Flag, Ski2Δ | *BY4741; hel2Δ:Leu2, EKD-Hel2-FLAG: Met, Ski2Δ::Kan* | This Study |
| Hel2 C67A-Flag, Ski2Δ | *BY4741; hel2D:Leu2, EKD-Hel2 C64,67A-FLAG: Met, Ski2Δ::Kan* | This Study |
| uS3, Ski2Δ | *BY4741, US3-WT::His, SKI2D::KAN* | This Study |
| uS3-K212R, Ski2Δ | *BY4741, uS3-K212R::His, Ski2Δ::Kan* | This Study |
| uS10, Ski2Δ | *BY4741, uS10-WT::His, Ski2Δ::Kan* | This Study |
| uS10 (K6,8R), Ski2Δ | *BY4741, uS10-K6,8R::His, SKI2D::Kan* | This Study |
| Gcn4-HA | *BY4741; GCN4-HA: Kan* | This Study |
| Hel2Δ, Gcn4-HA | *BY4741; hel2Δ::Leu2, GCN4-HA: Kan* | This Study |
| Hel2-Flag, Gcn4-HA | *BY4741; hel2Δ::Leu2, EKD-Hel2-FLAG: Met, GCN4-HA: Kan* | This Study |
| Hel2 C67A-Flag, Gcn4-HA | *BY4741; hel2Δ::Leu2, EKD-Hel2 C64,67A-FLAG: Met, GCN4-HA: Kan* | This Study |
| uS3, Gcn4-HA | *BY4741; uS3-WT::His, GCN4-HA: Kan* | This Study |
| uS3-K212R, Gcn4-HA | *BY4741; uS3-K212R::His, GCN4-HA: Kan* | This Study |
| uS10, Gcn4-HA | *BY4741; uS10-WT::His, GCN4-HA: Kan* | This Study |
| uS10-K6,8R, Gcn4-HA | *BY4741, uS10-K6,8R::His, GCN4-HA: Kan* | This Study |

## **Table2: Plasmids**

| **Plasmid Name** | **Description** | **Source** |
| --- | --- | --- |
| pRP469-PGK1 | PGK1 reporter | (1) |
| pRP1251-PGK1-SL | PGK1-SL reporter | (1) |
| pRP469-(CGA)_12_ | PGK1-(CGA)_12_ reporter | (2) |
| pRP469-(AAA)_12_ | PGK1-(AAA)_12_ reporter | (2) |
| pAG-426-gpd-ccdb | Empty plasmid for Hel2 | (3, 4) |
| pAG-Hel2 | pAG-426-Hel2 | This Study |

## **Table3: Primers**

| **Oligo Name** | **Sequence** | **Used for** |
| --- | --- | --- |
| Hel2::Leu2-F | CGA AAA AAT AGT GGC TAT ACT TCT TTT CAA GAA TTA GGG TTA ACT GTG GGA ATA CTC AGG | Hel2 deletion |
| Hel2::Leu2-F1 | GAT CCA TAA AGT GCA TTT TGA TAC AGT CTC TTT CGT CGA AAA AAT AGT GGC TAT ACT TCT | Hel2 deletion |
| Hel2::Leu2-R | AAT GCT ATT GTC AGT TAC AGG TTA GAA ATA TAT TTC CAA TTG ACT TAA ACT CCA TCA AAT | Hel2 deletion |
| Hel2::Leu2-R1 | TTT CTT TAT CGG AAT TTT ATT TAA GAC TTT CAT TTC TCT AAT GCT ATT GTC AGT TAC AGG | Hel2 deletion |
| Hel2-CHK-F | TTT CAT ACT CGC GGT CAC TC | Hel2 deletion check |
| Hel2-CHK-R | TAA CGC AGA CGT CTG AGC AG | Hel2 deletion check |
| Hel2-tag-F | AAG AAA GGC AAA CAA AAG CAG CTG TTA TTC CAC ATT GGT GTA GGG GGA GGC GGG GGT GGA | Tagging Hel2 with Flag |
| Hel2-tag-R | TTA AGA CTT TCA TTT CTC TAA TGC TAT TGT CAG TTA CAG GGA ATT CGA GCT CGT TTA AAC | Tagging Hel2 with Flag |
| Hel2 C64-67-F | CTG ATG AAG AAA ATG AAT TAT GTG TAA TTG CTG CGC GCA AGT TAA CAT ACG | Mutation of conserved cysteine residue (C67A) |
| Hel2 C64-67-R | CGT ATG TTA ACT TGC GCG CAG CAA TTA CAC ATA ATT CAT TTT CTT CAT CAG | Mutation of conserved cysteine residue (C67A) |
| HEL2Turbo-EKD-1-F | TTA GAA GTG TCA ACA ACG TAT CTA CCA ACT GGT ACC CTA TGA AGA AGT AGA CGG AGA ATG | Construction of integrating plasmid |
| HEL2Turbo-EKD-1-R | GCG TTC AGC AGC GGG ATT CCT CCA CCC CCG CCT CCC CCT ACA CCA ATG TGG AAT AAC AGC | Construction of integrating plasmid |
| TurboHel2-EKD-F | CAG CTG TTA TTC CAC ATT GGT GTA GGG GGA GGC GGG GGT GGA GGA ATC CCG CTG CTG AAC | Construction of integrating plasmid |
| TurboHel2-EKD-R | TCT AAT GCT ATT GTC AGT TAC AGG TTA GAA ATA TAT TTC CAA TTA GGT GCT GTC CAG GCC | Construction of integrating plasmid |
| HEL2Turbo-EKD-2-F | CCA ACC CCC TGC TGG GCC TGG ACA GCA CCT AAT TGG AAA TAT ATT TCT AAC CTG TAA CTG | Construction of integrating plasmid |
| HEL2Turbo-EKD-2-R | AAG GGC TGC AGT GCT AAG CTT CCA AAC TAC CAT AGG AAC TAA TCA ATA ATT TGA CTA CTC | Construction of integrating plasmid |
| EKD-HEL2-F | ACA ATG CTA AAA GAG TAG TCA AAT TAT TGA TTA GTT CCT ATG GTA GTT TGG AAG CTT AGC | Construction of integrating plasmid |
| EKD-HEL2-R | TTT AGC AAA AAC TTT CCA TTC TCC GTC TAC TTC TTC ATA GGG TAC CAG TTG GTA GAT ACG | Construction of integrating plasmid |
| EKD-Hel2-seq-1 | GTT TCA TAC TCG CGG TCA C | Hel2 insertion check in integrating plasmid |
| EKD-Hel2-seq-2 | CCT TTT TCA GAA ATA ACG CAG AC | Hel2 insertion check in integrating plasmid |
| Hel2-pAG-F | TAG AAC TAG TGG ATC CCC CAT CAC AAG TTT GTA CAA AAA AAT GAG CGA ATC AGT GAA AGA | Construction of pAG-426-Hel2 |
| Hel2-pAG-R | TGA ATG TAA GCG TGA CAT AAC TAA TTA CAT GAC TCG AGC TAC TTG TCA TCG TCA TCC TTG | Construction of pAG-426-Hel2 |
| pAG-Hel2-F | ATG ACA TCG ACT ACA AGG ATG ACG ATG ACA AGT AGC TCG AGT CAT GTA ATT AGT TAT GTC | Construction of pAG-426-Hel2 |
| pAG-Hel2-R | GTG TCG GAG TAA CGT TTT CTT TCA CTG ATT CGC TCA TTT TTT TGT ACA AAC TTG TGA TGG | Construction of pAG-426-Hel2 |
| Ski2:: kan F | ACTCACAAAATTTACTGTACTAATACTAATTTATCCGCTAGGGATAACAGGGTAATATAG | Ski2 deletion |
| Ski2::Kan F1 | TGCCACATAGTTCTTTCCGATATGAACAACCTAACTCACAAAATTTACTGTACTAATAC | Ski2 deletion |
| Ski2::Kan R | TATAAACATGACTCACATTGAGAATAAATGAGCTCTCTTAACTTCGCATCTGGGCAGATG | Ski2 deletion |
| Ski2::Kan R1 | GTGTGTGTGTGCAATAAGAGTTCGAAAACATTAACTTTTATAAACATGACTCACATTGAG | Ski2 deletion |
| Ski2 CHK F | CGCTGCACACAACGGGTTTTCC | Ski2 deletion check |
| Ski2 CHK R | CTTCTTCATCACCCGCTCTAGC | Ski2 deletion check |
| GCN4-HA-F | GAA AAT GAG GTT GCC AGA TTA AAG AAA TTA GTT GGC GAA CGC GGG GGT GGA TAC CCA TAC | Tagging GCN4 with HA |
| GCN4-HA-R | AAT GAA ATA AAA AAT ATA AAA TAA AAG GTA AAT GAA ATT GAC TTA AAC TCC ATC AAA TGG | Tagging GCN4 with HA |
| GCN4-HA-F1 | AAG GTT GAA GAA TTG CTT TCG AAA AAT TAT CAC TTG GAA AAT GAG GTT GCC AGA TTA AAG | Tagging GCN4 with HA |
| GCN4-HA-R1 | TAA GTG AAT GTA TCT ATT TCG TTA TAC ACG AGA ATG AAA TAA AAA ATA TAA AAT AAA AGG | Tagging GCN4 with HA |
| GCN4-CHK-F | TCT CGT GCG AGA AAG TTG C | GCN4 HA tag check |
| GCN4-CHK-R | CGG CAG ATT ATA AAT GCG TGG | GCN4 HA tag check |
| 5’PGK Probe | AACTGGAGCCAAAGAGTATTTTTCGTTTCTTTCACCGTTTGGTCTACCCAAGTGAGAAGC | Northern Blotting |
| SCR1 Probe | GTCTAGCCGCGAGGAAGG | Northern Blotting |

## **References**

1. Doma, M. K., andParker, R. (2006) Endonucleolytic cleavage of eukaryotic mRNAs with stalls in translation elongation Nature **440**, 561-564 10.1038/nature04530

2. Simms, C. L., Yan, L. L., andZaher, H. S. (2017) Ribosome Collision Is Critical for Quality Control during No-Go Decay Mol Cell **68**, 361-373 e365 10.1016/j.molcel.2017.08.019

3. Alberti, S., Gitler, A. D., andLindquist, S. (2007) A suite of Gateway cloning vectors for high-throughput genetic analysis in Saccharomyces cerevisiae Yeast **24**, 913-919 10.1002/yea.1502

4. Yan, L. L., Simms, C. L., McLoughlin, F., Vierstra, R. D., andZaher, H. S. (2019) Oxidation and alkylation stresses activate ribosome-quality control Nat Commun **10**, 5611 10.1038/s41467-019-13579-3
